# Assessment of Liver Fibrosis Using Non-invasive Screening Tools in Individuals With Diabetes Mellitus and Metabolic Syndrome

**DOI:** 10.7759/cureus.22682

**Published:** 2022-02-28

**Authors:** Namitha Shaji, Abhishek Singhai, Radha Sarawagi, Abhijit P Pakhare, V N Mishra, Rajnish Joshi

**Affiliations:** 1 Internal Medicine, All India Institute of Medical Sciences (AIIMS), Bhopal, IND; 2 Radiodiagnosis, All India Institute of Medical Sciences (AIIMS), Bhopal, IND; 3 Community and Family Medicine, All India Institute of Medical Sciences (AIIMS), Bhopal, IND; 4 Internal Medicine, All India Institute of Medical Sciences, Bhopal, Bhopal, IND

**Keywords:** hepatic fibrosis, metabolic disorder, diabetes type 2, cirrhosis, nonalcoholic fatty liver disease (nafld)

## Abstract

Introduction: Despite the rising prevalence of liver fibrosis and its potentially life-threatening complications, there are currently no recommendations or guidelines to screen individuals with diabetes mellitus (DM) or high body mass index (BMI) for non-alcoholic fatty liver disease (NAFLD)/non-alcoholic steatohepatitis (NASH). This is mainly due to the uncertain performance and feasibility of presently available screening tools. This research was carried out to assess the diagnostic accuracy of non-invasive screening tools in predicting liver fibrosis in individuals with DM and metabolic syndrome.

Methods: For this study, 140 patients with DM and metabolic syndrome were identified between March 2020 and October 2021. Liver stiffness measurement by point shear wave elastography was considered the gold standard in our study. Five non-invasive scores such as aspartate aminotransferase/alanine aminotransferase (AST/ALT) ratio, aspartate aminotransferase to platelet ratio index (APRI) score, fibrosis-4 (FIB-4) index, BARD score, and NAFLD fibrosis score were determined in all of the participants. Using receiver operator characteristic (ROC) curve analysis, sensitivity, specificity, both negative predictive value (NPV) and positive predictive value (PPV) were calculated for each of these scores. The area under the ROC curve (AUROC) was used to calculate the diagnostic accuracy of these scores.

Results: Of the 507 individuals screened, 140 were enrolled for the study. Among the 140 participants, 83 were male (59.29%), 30 (21.43%) had liver fibrosis as per liver stiffness measurement by point shear wave elastography, and 110 (78.57%) did not have fibrosis. The mean age and mean BMI were 54.53±12.42 and 27.37±2.73 respectively in the ‘Fibrosis’ group and 56.20 ±11.76 and 27.10±4.22 in the ‘No fibrosis’ group. The major finding of our study was that all these scores had relatively high NPV (>85 %) for predicting liver fibrosis in our cohort. The AST/ALT ratio had the highest NPV (90.28%) followed by APRI Score (88.94%). The AUROC for FIB-4 Score, NAFLD-fibrosis score, APRI score, AST/ALT ratio, and BARDd score were 0.6669, 0.657, 0.655, 0.637 and 0.599, respectively. The FIB-4 index (p=0.005) had the highest AUROC, followed by the NAFLD-fibrosis score (p =0.009). But all the scores had relatively low specificity (<60 %), PPV (<35 %), and accuracy (<63 %).

Conclusion: The FIB-4 index and NAFLD-fibrosis score can be used reliably to exclude liver fibrosis in individuals with DM and metabolic syndrome in the Indian population, but may not be useful in accurately diagnosing liver fibrosis. Utilization of these non-invasive and cost-effective screening tools in routine practice may have promising results in predicting liver fibrosis in ‘at risk’ populations.

## Introduction

Non-alcoholic fatty liver disease (NAFLD) is defined as the presence of ≥ 5% hepatic steatosis either on imaging or on liver histology after excluding secondary causes of fat accumulation in the liver (e.g., significant alcohol consumption, certain medications, and other medical conditions) [1]. In India, the prevalence of NAFLD in the general population is around 9% to 32 %. The prevalence varies from region to region, ranging from 44.1% in western states of India to 72.4% in the northern parts [2].

Approximately 30% to 40% of patients with NAFLD, progress to the advanced end of the chronic NAFLD spectrum and non-alcoholic steatohepatitis (NASH). After 10 years, 10% to 30% of NAFLD patients may eventually progress to cirrhosis and hepatocellular carcinoma [3]. Studies have reported that NAFLD-related complications are not only limited to the liver but is also associated with a high risk of extra-hepatic complications which may contribute to morbidity and mortality, such as extra-hepatic cancers (colorectal cancers), chronic kidney disease (CKD), and cardiovascular disease (CVD). Cardiovascular disease is estimated to be the most predominant cause of mortality in NAFLD patients [4].

The obesity epidemic has begun to rise, and individuals are victims of this disease even from their childhood. Obesity at a young age promotes the premature development of metabolic syndrome, which in turn leads to NAFLD early in life. Non-alcoholic fatty liver disease is considered to be the hepatic manifestation of metabolic syndrome [3]. Type 2 diabetes mellitus (DM) is another disease entity that contributes to the incidence and progress of NAFLD [5]. The prevalence of NAFLD varies according to the presence or absence of comorbidities. It can rise to as high as 80% to 90% in individuals who are obese, up to 60% in dyslipidaemia patients, and up to 30% to 50% in individuals with diabetes mellitus. Current studies have shown that the threshold for the development of NAFLD varies with ethnicity. It was noticed that individuals of Asian decent develop NAFLD at a lower body mass index (BMI) [6].

Since attending to NAFLD and its potential complications is becoming a growing burden on healthcare systems, the need arises for screening at-risk individuals for the same. Liver biopsy remains the gold standard for diagnosing liver fibrosis, but it is poorly accepted in the general population due to various reasons such as cost, invasiveness and associated complications. This prompts us to identify and develop easier, more accessible, non-invasive tools for screening such individuals so that the disease can be picked up at an early stage where treatment strategies may prove to be beneficial [7].

As of now, numerous non-invasive tools such as aspartate aminotransferase/alanine aminotransferase (AST/ALT) ratio, aspartate aminotransferase to platelet ratio index (APRI), fibrosis-4 (FIB-4) index, BARD score, NAFLD-fibrosis score along with imaging modalities for liver stiffness measurements through shear wave elastography, transient elastography and magnetic resonance elastography, have gained popularity. We aimed to assess the diagnostic accuracy of non-invasive screening tools in predicting liver fibrosis in individuals with DM and metabolic syndrome. Our study hopes to answer this research question - Among adults with DM and metabolic syndrome, are non-invasive screening tools (AST/ALT ratio, APRI score, FIB-4 index, BARD score and NAFLD-fibrosis score) more accurate than point shear wave liver elastography in the accurate diagnosis of liver fibrosis.

## Materials and methods

Design and ethics

We performed a hospital-based cross-sectional diagnostic accuracy study to address our research question. The study design was approved by the Institutional Human Ethics Committee of AIIMS, Bhopal (IRB No. IHECPGRMD030). All the study participants underwent the study procedure after written informed consent.

Participants

We screened all adults above the age of 18 years who presented to the outpatient department (OPD) and wards for our study. We included individuals with DM as per current Americans with Disabilities Act (ADA) 2021 guidelines, and metabolic syndrome as per the 2005 revised National Cholesterol Education Program (NCEP) Adult Treatment Program (ATP) III guidelines. We excluded individuals with overt cirrhosis, positive hepatitis B surface antigen, positive antibody against hepatitis C virus, congestive hepatopathy, active malignancy [8], and secondary causes of fatty liver. We also excluded pregnant women, individuals using/having used hepatotoxic drugs in the last six months (over 4 g/day of acetaminophen, methotrexate, nitrofurantoin, and rifampicin). Also, men who have consumed >140g and women who have consumed >70 g of alcohol per week for at least 10 years were excluded. The sample size was calculated using the formula, n=[4 x Sn x (1-Sn)]/[(Error)2 x Prevalence], where the error was 10% of sensitivity expressed as proportion, prevalence (of liver fibrosis in patients with diabetes and metabolic syndrome) expressed as proportion was taken as 50% (range is 34% to 74%), and Sn was sensitivity expressed as proportion. The sensitivity of each non-invasive score was taken and the sample size was calculated for each. The highest sample size was taken for the study. By taking the sensitivity of APRI and NAFLD fibrosis score which was around 77%, the sample size came to 140.

Procedures

**Figure 1:**
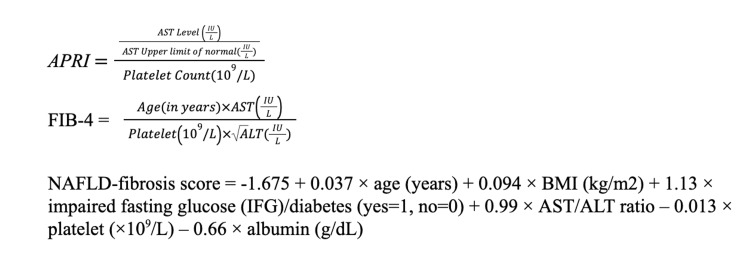
Formulae to calculate APRI, FIB-4, and NAFLD-fibrosis score APRI: Aspartate aminotransferase to platelet ratio index, FIB-4: Fibrosis 4, NAFLD: Nonalcoholic fatty liver disease, AST: Aspartate aminotransferase, ALT: Alanine transaminase

Screening of individuals for enrolment in the study was conducted from March 2020 to September 2021. All the patients satisfying the inclusion criteria were evaluated for eligibility criteria and approached for participation in the study. Informed consent was obtained. Eligible and consenting participants were included in the study. A structured interview of all participants was performed and a brief questionnaire to obtain demographic, clinical, and DM and metabolic syndrome-related information was administered. Study participants were categorized in BMI class as per WHO 2009 Asian-Indian specific guidelines. The most recent (within the previous three months) biochemical investigations from previous records, including reports of complete blood count, liver function tests, renal function tests, serum lipid profile, fasting blood sugar were taken into account. The non-invasive scores were calculated using appropriate software with the data collected. The formulae for calculating the non-invasive scores are given in Figure 1.

The BARD score was calculated using three variables and by assigning points to each, BMI>28 kg/m2 (1 point if yes), AST/ALT ratio≥0.8 2 points if yes), and the presence of DM (1 point if present). Point shear wave liver elastography was done by an expert blinded radiologist using Siemens Acuson S-3000 (Siemens Medical Solutions, Mountain View, CA, USA) using a curvilinear probe. The values of the non-invasive scores and reports of point shear wave liver elastography were analyzed.

Statistical analysis

Statistical analysis was carried out using statistical packages for the social sciences (SPSS) version 22 (IBM SPSS Statistics, Armonk, NY, USA). Continuous and categorical variables were expressed as mean ± standard deviation and percentages, respectively. Two-sided p-values were considered statistically significant at a p-value<0.05.

A Chi-square test was done to see the association of clinical symptoms with fibrosis/non-fibrosis. An Independent t-test was done to compare test variables and lab parameters between fibrosis/no fibrosis. Receiver operating characteristic (ROC) curves with area under the ROC (AUROC) was done for AST/ALT ratio, APRI score, FIB-4 index, BARD score, and NAFLD-fibrosis score. Sensitivity, specificity, PPV, NPV, likelihood ratio, and accuracy were calculated for AST/ALT ratio, APRI score, FIB-4 index, BARD score and NAFLD-fibrosis score.

## Results

**Figure 2:**
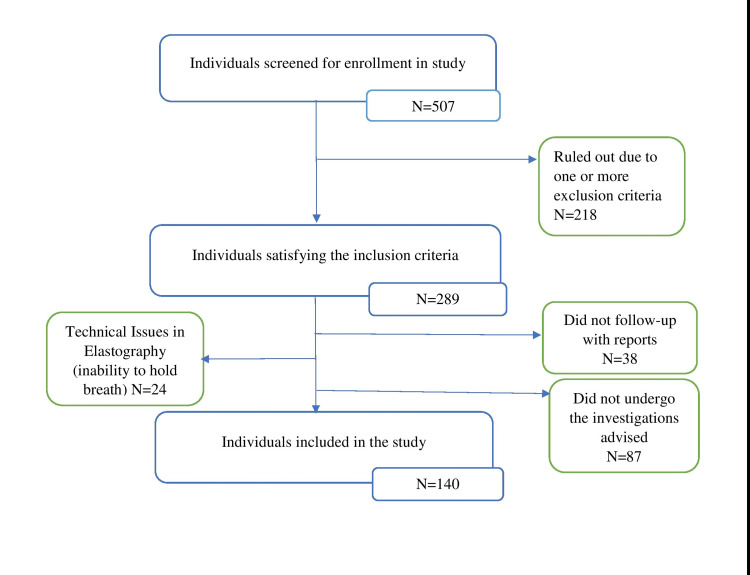
Study flowchart N: Number

Of the 507 individuals screened, 140 were enrolled for the study. Among the 140 participants, 83 were male (59.29%), 57 (40.71%) were female, 30 (21.43%) had liver fibrosis as per liver stiffness measurement by point shear wave elastography, and 110 (78.57 %) did not have fibrosis (Figure 2). In the ‘Fibrosis’ group (48.2 %) as well as the ‘No fibrosis’ group (40 %), the duration since diagnosis of DM in majority of the study participants was between one to five years (Table 1). There was no significant correlation between fibrosis and duration since the diagnosis of diabetes mellitus (p=0.711). In the ‘No fibrosis’ group (n=110), 96 87.3%) had hypertension, 67 (60.0%) had dyslipidemia, and 11 (10%) had comorbidities other than hypertension or dyslipidemia (chronic kidney disease and coronary artery disease). In the ‘Fibrosis’ group (n =30), 27 had hypertension (90%), 18 (60 %) had dyslipidemia, and one had comorbidity (chronic kidney disease) other than hypertension or dyslipidaemia.

**Table 1:** Characteristics of the study population Plus-minus values are mean ± SD unless otherwise indicated. *: Difference was assessed using an independent t-test with a p>0.05 considered not significant; α: This was assessed by Chi-square test, p>0.05 not significant; η: Significant, p <0.05; BMI: Body mass index, OHA: Oral hypoglycemic drugs

S.No	Variables		No Fibrosis (n=110)	Fibrosis (n=30)	p-value
1	Age (Years)*		56.20 ±11.76	54.53±12.42	0.511
2	Males ^α^		63 (57.3%)	20 (66.7%)	0.356
3	Females^ α^		47 (42.7%)	10 (33.3%)	
4	Height (cm)*		157.31±26.47	152.84±41.29	0.578
5	Weight (kg)*		70.89±12.46	73.33±8.41	0.214
6	BMI (kg/m^2 ^)*		27.10±4.22	27.37±2.73	0.675
	Normal ^α^		23 (20.9%)	2 (6.7%)	0.286
	Pre-obese/Overweight		14 (12.7%)	5 (16.7%)	
	Obese Class 1		52 (47.3%)	18(60%)	
	Obese Class 2		21 (19.1%)	5 (16.7%)	
7	Waist Circumference (inches)				
	<35 (F)/<40 (M)		35 (31.8%)	7 (23.3%)	0.369
	>35 (F)/>40 (M)		75 (68.2%)	23 (76.7%)	
8	Current Antidiabetic Medications				
	Insulin	Yes	15(13.6)	0(0)	0.032 ^η^
		No	95 (86.4%)	30 (100%)	
	OHA	Yes	100 (90.9%)	30(100)	0.087
		No	10 (9.1%)	0 (0%)	
9	Hypertension	Yes	96 (87.3%)	27 (90%)	0.685
		No	14 (12.7%)	3 (10%)	
10	Dyslipidemia	Yes	67 (60.9%)	18 (60%)	0.928
		No	43 (39.1%)	12 (40%)	
11	Other Comorbidities	Yes	11 (10%)	1 (3.3%)	0.248

There was no significant correlation between platelet counts, serum triglyceride levels, high-density lipoprotein (HDL) levels, fasting blood sugar, and glycated haemoglobin (HbA1c) levels with fibrosis (Table 2).

**Table 2:** Laboratory parameters of the study population Plus-minus values are mean ± SD unless otherwise indicated. HDL: High-density lipoprotein, VLDL: Very low-density lipoprotein, FBS: Fasting blood sugar, PPBS: Postprandial blood sugar, HbA1c: Glycosylated hemoglobin

S.No	Lab Parameters	No Fibrosis (N=110)	Fibrosis (N=30)	p-value
1	Platelet (x10^9^ /L)	2.80±0.79	2.53±0.79	0.106
2	Triglyceride (mg/dL)	150.4±88.79	133.8±68.89	0.280
	< 150	54 (49.1%)	18 (60%)	0.289
	>150	56 (50.9%)	12 (40%)	
3	HDL (mg/dL)	43.49±14.40	44.47±18.67	0.791
	< 50 (F) / <40 (M)	70 (63.6%)	20 (66.7%)	0.759
	≥ 50 (F) / ≥ 40(M)	40 (36.4%)	10 (33.3%)	
4	VLDL(mg/dL)	37.64±31.28	31.21±18.54	0.159
5	FBS (mg/dL)	158.66±58.87	158.61±75.67	0.997
	<130	38 (34.5%)	12 (40%)	0.58
	>130	72 (65.5%)	18 (60%)	
6	PPBS (mg/dL)	237.28±80.25	239.96±98.23	0.891
	≤180	23 (20.9%)	8 (26.7%)	0.501
	>180	87 (79.1%)	22 (73.3%)	
7	HbA1c (%)	7.92±1.53	8.30±2.30	0.406
	<7	35 (31.8%)	13 (43.3%)	0.239
	>7	75 (68.2%)	17 (56.7%)	

The mean values of the non-invasive markers of liver fibrosis (AST/ALT ratio, APRI score, FIB-4 index, BARD score and NAFLD-fibrosis score) in the participants are enumerated in Table 3.

**Table 3:** Non-invasive scores for liver fibrosis AUROC: Area under the receiver operating characteristics, PPV: Positive predictive value, NPV: Negative predictive value

Test Variable	Cut-off	AUROC	Sensitivity (%)	Specificity (%)	PPV (%)	NPV (%)	Accuracy (%)	p-Value
AST: ALT Ratio	0.8	0.637	76.67	59.09	33.82	90.28	62.86	0.029
APRI Score	0.26	0.655	80	41.82	27.27	88.46	50	0.108
FIB-4 Score	0.97	0.669	73.33	44.55	26.51	85.96	50.71	0.007
BARD Score	2	0.599	76.67	40.91	26.14	86.54	48.74	0.103
NAFLD-Fibrosis Score	1.61	0.657	70	50	27.63	85.94	54.29	0.012

**Figure 3:**
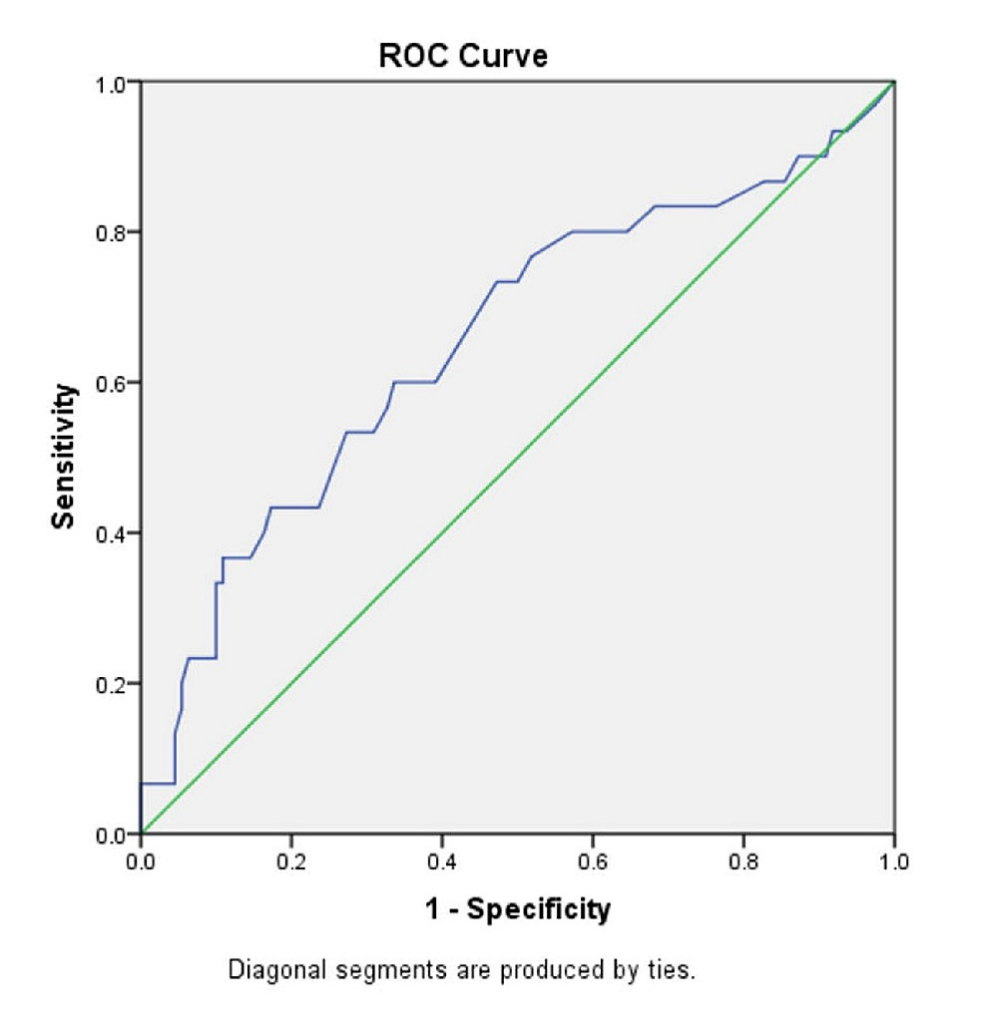
ROC curve for AST/ALT ratio ROC curve: Receiver operating characteristic curve, AST: Aspartate aminotransferase, ALT: Alanine transaminase

**Figure 4:**
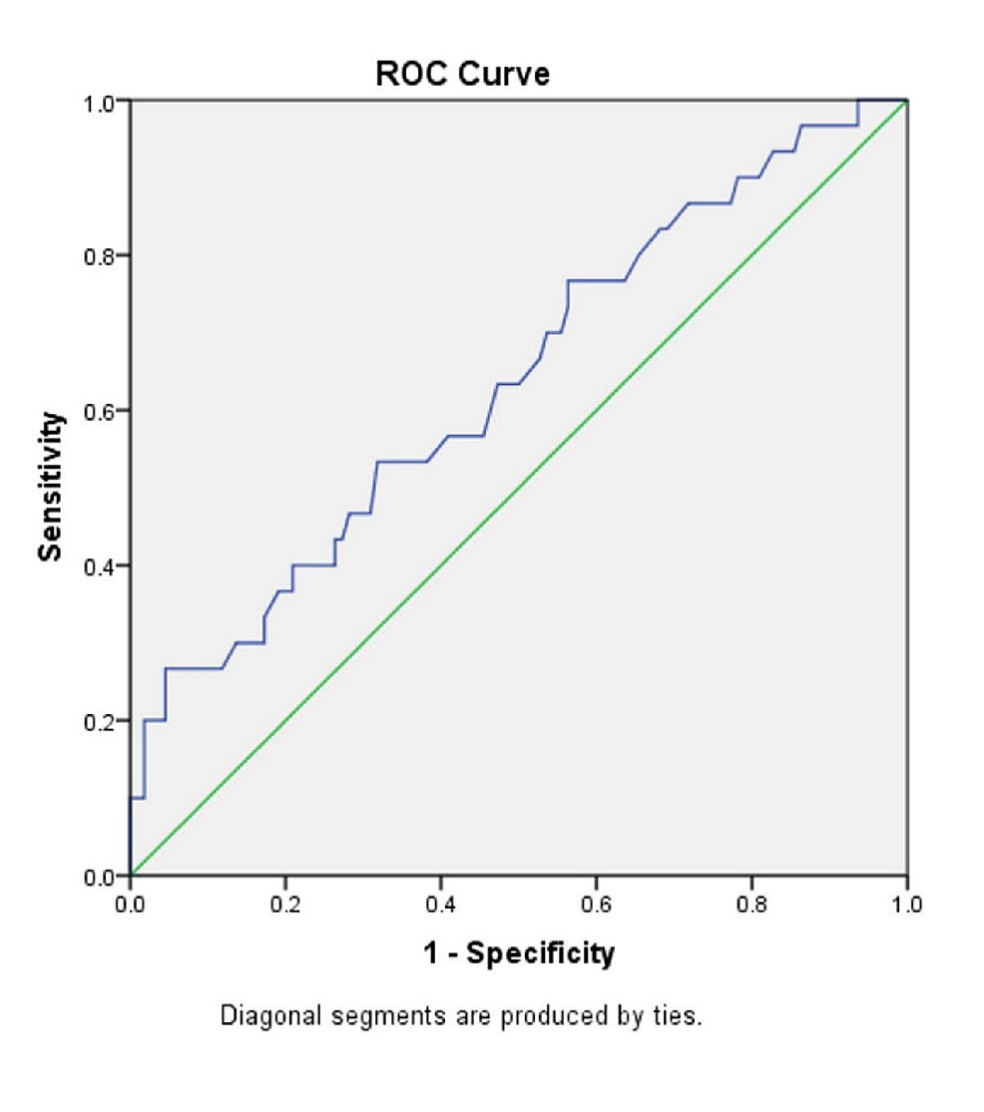
ROC curve for APRI score ROC curve: Receiver operating characteristic curve, APRI: Aspartate aminotransferase to platelet ratio index

**Figure 5:**
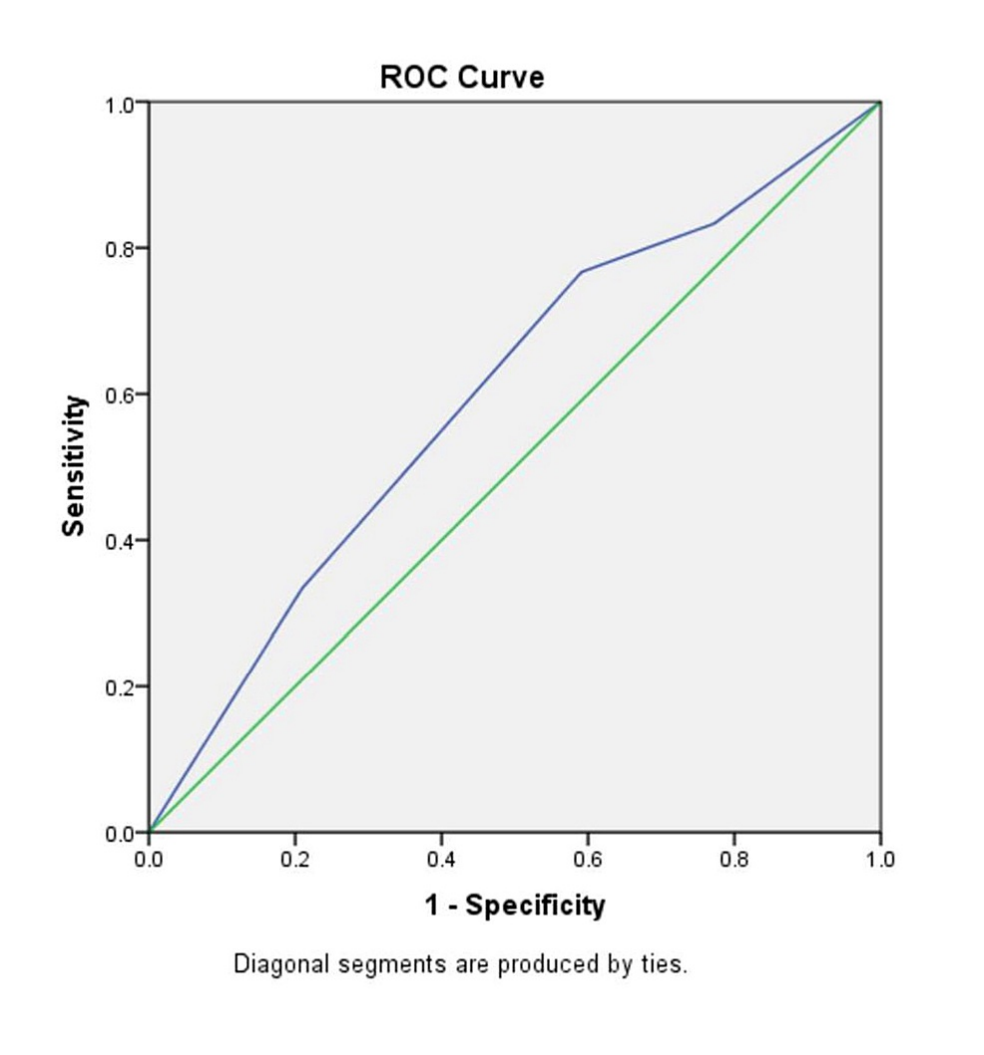
ROC curve for FIB-4 index ROC: Receiver operating characteristic curve, FIB-4: Fibrosis-4

**Figure 6:**
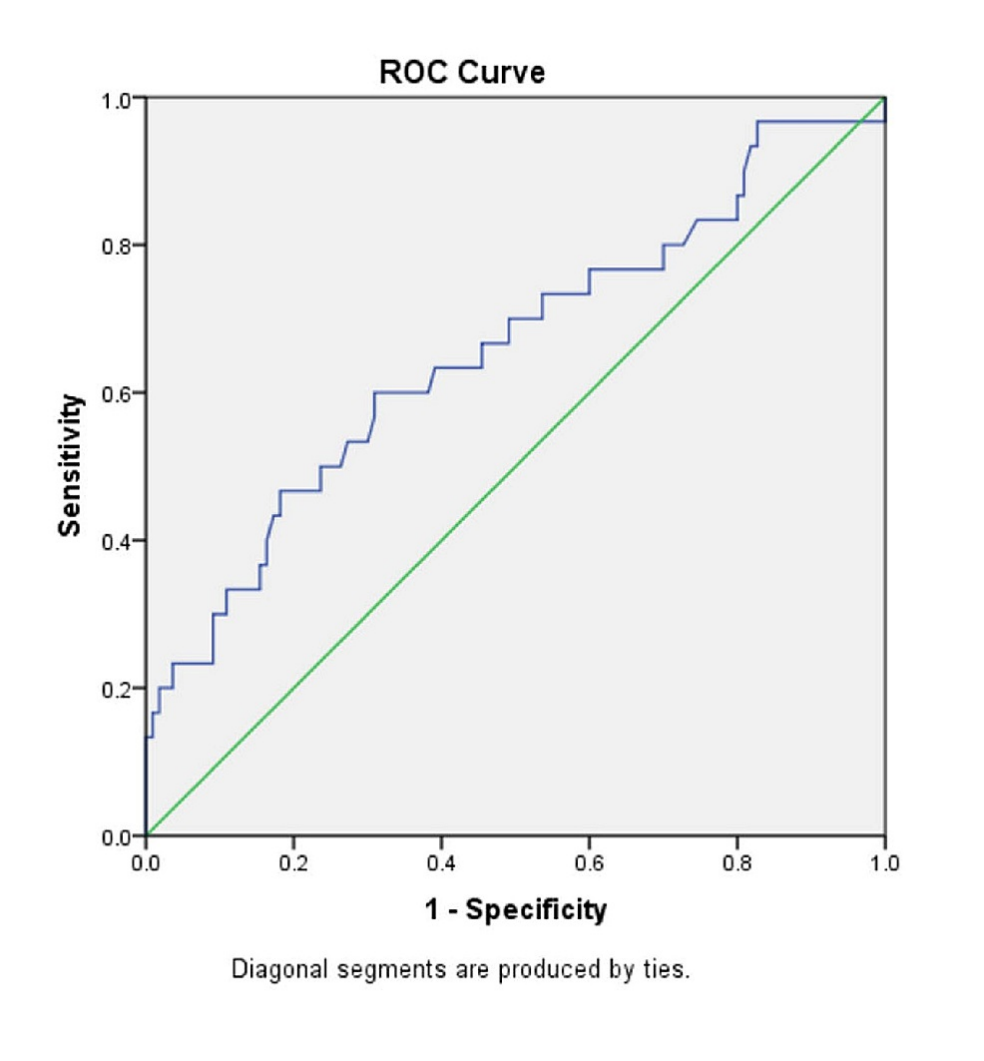
ROC curve for NAFLD fibrosis score ROC: Receiver operating characteristic curve, NAFLD: Nonalcoholic fatty liver disease

**Figure 7:**
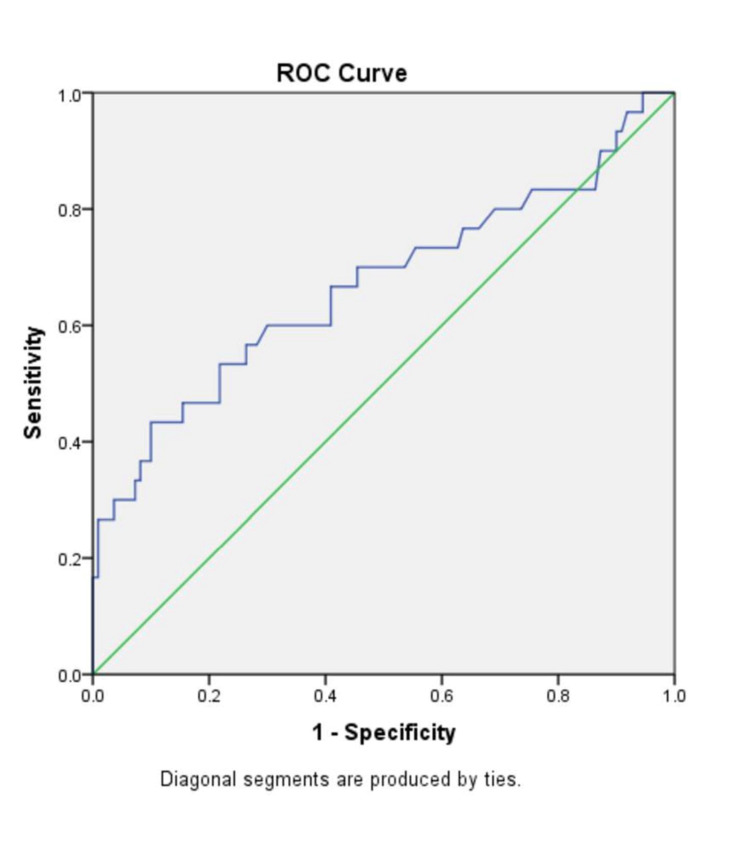
ROC curve for BARD score ROC: Receiver operating characteristic curve

**Figure 8:**
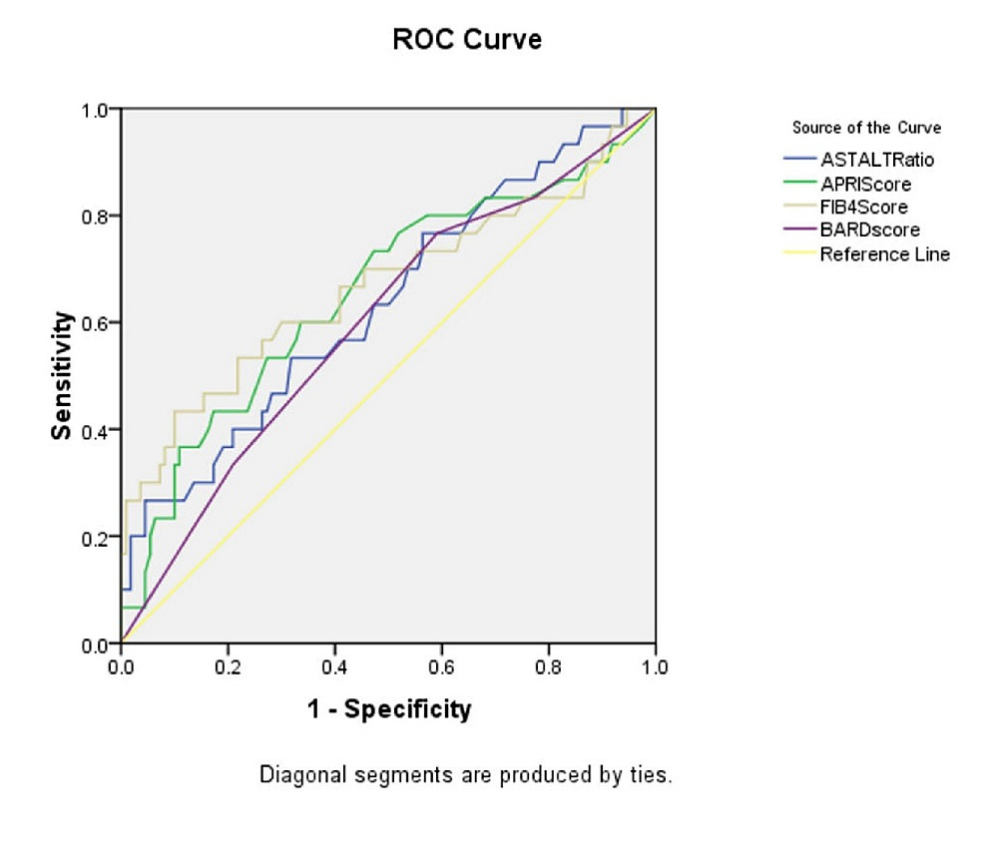
ROC curve for the non-invasive markers of liver fibrosis (AST/ALT ratio, APRI score, FIB-4 index, BARD score and NAFLD-fibrosis score) ROC: Receiver operating characteristic curve, AST: Aspartate aminotransferase, ALT: Alanine transaminase, APRI: Aspartate aminotransferase to platelet ratio index, FIB-4: fibrosis-4, NAFLD: Nonalcoholic fatty liver disease

In the ‘No fibrosis’ group and the ‘Fibrosis’ group, the mean (± SD) AST/ALT ratio were 0.89 ± 0.30 and 1.24 ± 0.81, respectively (p-value=0.029); the mean (± SD) APRI score was 0.38 ± 0.42 and 0.80 ± 1.36 (p-value=0.108); the mean (± SD) FIB-4 index was 1.16 ± 0.57 and 2.15 ± 1.85 (p-value=0.007); the BARD score mean (± SD) was 2.57 ± 1.06 and 2.93 ± 1.04, (p-value=0.103); the mean (± SD) NAFLD-fibrosis score was -1.54 ± 1.06 and -0.61 ± 1.81 (p-value=0.012). There were significantly higher mean values of AST/ALT ratio, FIB-4 index and NAFLD-fibrosis score in the individuals with fibrosis as compared to those with no fibrosis (p<0.05 in all). There was no significant difference between the two groups in the APRI score and BARD score. To compare the diagnostic accuracy of each of the non-invasive markers, receiver operating characteristic (ROC) curves were constructed for the non-invasive marker values (Figures 3-8).

## Discussion

In our study, we assessed the diagnostic accuracy of certain non-invasive scores currently being used for diagnosing liver fibrosis (AST/ALT ratio, APRI score, FIB-4 index, BARD score, NAFLD-fibrosis score. We analyzed these scores in comparison with point shear wave elastography.

In shear wave elastography, shear waves are generated to create dynamic stress, and the physical tissue displacement parallel to the applied stress is measured. The speed of the shear wave is studied to give an idea of the elasticity of the tissue [9]. The principle of point shear wave elastography, which was the modality used in this study, is to induce tissue displacement at a single focal location in the normal direction. Here, the tissue displacement itself is not measured but by absorbing acoustic energy, a portion of the longitudinal waves generated are converted into shear waves. The unit of shear wave speed is m/s (meter/second), which is then converted to kPa (kilo Pascal). Gharibvand et al. proposed that the sensitivity and specificity of shear point wave elastography was 81.76% and 77.01% for fibrosis stage F2, 90.20% and 78.40% for fibrosis stage F3 and 89.53% and 94.38% for fibrosis stage F4 [10]. Thus, shear point wave elastography was a useful and effective diagnostic tool for assessing liver fibrosis in chronic liver disease with a diagnostic accuracy comparable to that of liver biopsy along with the added advantage of being a non-invasive modality [10].

Factors such as age greater than 45 years, obesity, hypertension, elevated AST/ALT ratio and hyperlipidemia are found to be associated with increased risk of developing fibrosis and increased chance of progression of fibrosis to cirrhosis [11]. Association between BMI and liver fibrosis is controversial with some studies suggesting a significant correlation while others deny it [12,13]. There was no significant correlation between BMI or age with fibrosis in our study.

No significant correlation could be found between fibrosis and sex, duration since diagnosis of DM (p=0.711), and the presence of comorbidities (hypertension, dyslipidaemia and chronic kidney disease). This was in contrast to the study by Byrne et al., where there was a significant association and causal link between CKD and NAFLD and it was concluded that the presence of NAFLD in an individual is a driving factor for developing CKD [14]. Concerning coronary artery disease, Kirby et al. concluded that there was no direct relationship between the presence of CVD and liver fat accumulation [15]. In contrast, the results of a study by Montemezzo et al. proposed a strong association between CVD and NAFLD [16].

In our study, the AST/ALT ratio had an AUROC of 0.637 and at a cut-off 0.8, it had an NPV of 90.28 % while the PPV was 33.82%, and the diagnostic accuracy was 62.86%. As compared to the other scores in our study, AST/ALT ratio had the highest NPV and diagnostic accuracy. Though AST/ALT ratio has a low diagnostic accuracy of liver fibrosis, the fact that it has a high NPV ensures that it may be able to exclude liver fibrosis in our cohort.

The APRI score had been developed originally for assessing liver fibrosis in patients with chronic hepatitis C, where it could identify liver fibrosis with a high degree of accuracy [17]. But recently, its use in NAFLD has been studied, where it was proposed that APRI score is superior to AST/ALT ratio in detecting liver fibrosis in NAFLD [18]. Studies have assessed the usefulness of the APRI score in NAFLD and one study found that the APRI score had 27% sensitivity, 89% specificity, 37% PPV and 95% NPV. [19] Calès P et al. assessed the performance of APRI Score in a French cohort, which showed a sensitivity, specificity, PPV and an NPV of 66%, 90%, 72% and 87%, respectively and these were the highest sensitivity and PPV reported till now [19]. In comparison to these studies, our results with an APRI score cut-off of 0.26, showed a much higher sensitivity of 80% and an almost similar NPV of 88.46%, but a lower specificity of 41.82%, a lower PPV of 27.27%, and a diagnostic accuracy of 62.86%.

The FIB-4 index was also originally developed to assess the staging of liver fibrosis in chronic hepatitis C patients and has also been applied in patients with chronic hepatitis B [20]. It has now been validated in NAFLD patients as well. Two FIB-4 index cut-offs were found to differentiate between the presence of liver fibrosis (high cut-off of 2.67) and the absence (low cut-off of 1.3) of liver fibrosis. Shah et al. showed that with a high cut-off (>2.67), the FIB-4 index had a PPV of 80% to rule in advanced fibrosis, and with a low cut-off (<1.3) it could rule out advanced fibrosis with an NPV of 90% to 95%. The AUROC (0.80-0.86) for predicting advanced fibrosis was also highest for the FIB-4 index as compared to other non-invasive markers (AST/ALT ratio, NAFLD-fibrosis score and BARD score) [21]. A study by Mohamed et al. had similar results where the FIB-4 index had the highest AUROC curve (0.936) [22]. In their study, the FIB-4 index using the high cut-off (>2.67) had a sensitivity, specificity and a PPV of 63.2%, 93% and 75%, respectively. And using the low cut-off, it had a sensitivity, specificity and NPV of 89 %, 86.9% and 94.2%, respectively. In our study as well, the FIB-4 index had the highest AUROC of 0.669. The ROC derived cut-off was 0.97 and using this cut-off, the FIB-4 Index had a sensitivity of 73.3%, a specificity of 44.55%, a PPV of 26.51%, NPV of 85.96 %, and a diagnostic accuracy of 50.71%. The results suggest that the FIB-4 index may be used in daily practice for ruling out fibrosis (NAFLD) in individuals with DM and metabolic syndrome.

Harrison et al. developed the BARD score using three variables i.e., BMI>28 kg/m2, AST/ALT ratio≥ 0.8, and the presence of DM, as these variables were individually associated by univariate analysis for advanced fibrosis, with odds ratios of ≥2.4. The final score ranged from 0 to 4 points. A BARD score of 2 to 4 was associated with an odds ratio of 17 (95% CI, 9.2-31.9), NPV of 96 % and an AUROC of 0.81 for identifying advanced fibrosis [23]. But recent studies have shown that the AUROCs are lower (0.65 to 0.7) for BARD score. [21] In our study, the BARD score had the lowest AUROC (0.599). Using a cut-off 2, it had a sensitivity of 76.67%, a specificity of 40.91 %, a PPV of 26.14%, NPV of 86.54 %, and a diagnostic accuracy of 48.74%.

The NAFLD-fibrosis score (NFS) was developed by Angulo et al. to assess the stage of liver fibrosis in NAFLD and is independent of the ALT levels. Among APRI Score, the FIB-4 index and NAFLD-fibrosis score, the most extensive validation were received by NFS. The AASLD (American Association for the Study of Liver Diseases) currently recommends the use of NFS in diagnosing advanced fibrosis in NAFLD in routine clinical practice. The NFS was proposed to have two cut-offs, a high cut-off of 0.67 and a low cut-off of −1.455. A score of more than 0.67 indicates the presence of advanced fibrosis while a score of less than −1.455 rules out advanced fibrosis. One drawback of NFS is that approximately 25% to 30% of patients have a score between -1.455 to 0.676 and these patients are considered to have an intermediate score. The NFS was shown to have a sensitivity of 22%, a specificity of 100%, a PPV of 90% and an NPV of 93% [24]. Other studies have reported varying ranges of results with a sensitivity ranging from 22% to 78 % [13], specificity from 58% to 100 %, NPV ranging from 92% to 100 %, and a PPV ranging from 26% to 81% [25]. In our study, the NAFLD-fibrosis score had the second-highest AUROC (0.657) followed by the FIB-4 score. Using a cut-off of -1.61, the sensitivity was 70%, the specificity was 50%, the PPV was 27.63%, the NPV was 85.96% and diagnostic accuracy was 54.29%. This suggests that even though the NAFLD-fibrosis score was not able to accurately diagnose liver fibrosis in our study participants, it could be used for ruling out liver fibrosis in individuals with diabetes mellitus and metabolic syndrome.

When compared with existing literature, the sensitivity of the five scores in our study was relatively similar (70% to 80 %). But contrary to previous studies that suggested that APRI, FIB-4 and NFS had higher specificity (80% to 98%), the specificity for these scores in our study was in the range of 40% to 50%. The major finding of our study was that all these scores had relatively high NPV (>85 %) for diagnosing liver fibrosis in individuals with DM and metabolic syndrome, which was consistent with previous studies. The AST/ALT ratio had the highest NPV (90.28%) followed by the APRI score (88.94%). The AUROC was highest for FIB-4 score (0.669) followed by NAFLD-fibrosis score (0.657) similar to the study by Mohamed et.al [22] and Perez et al. [13]. In short, the non-invasive scores (AST/ALT ratio, APRI score, FIB-4 index and NAFLD-fibrosis score), especially FIB-4 index and NAFLD-fibrosis score can be used reliably in routine practice to exclude or rule out liver fibrosis in individuals with DM and metabolic syndrome. This study is one of the first few studies in our country to assess the accuracy of non-invasive screening tools for diagnosing liver fibrosis in individuals with DM and metabolic syndrome. The study participants were selected randomly from our OPD as well as medical wards. All values used for comparison are machine-generated values. The point shear wave liver elastography that was used as a gold standard in our study was done by blinded experts. All the non-invasive scores were calculated by appropriate software. The study used all the latest guidelines.

Our study does have some limitations. The first is the small sample size. More participants could have been included in the study to get a better idea of the reliability of these non-invasive screening tools. Second, point shear wave liver elastography was used as a gold standard to compare the performance of the non-invasive liver fibrosis markers instead of liver biopsy which is the recommended gold standard for diagnosing liver fibrosis.

## Conclusions

To conclude, the FIB-4 index and NAFLD-fibrosis score proved to be a reliable non-invasive tool to exclude liver fibrosis in individuals with diabetes mellitus and metabolic syndrome. But the non-invasive scores (AST/ALT ratio, APRI score, FIB-4 index, BARD score, NAFLD-fibrosis score) lack the required specificity and accuracy. Hence, it is not useful in definitively and accurately diagnosing liver fibrosis.
